# Neural Collaborative Filtering with Ontologies for Integrated Recommendation Systems

**DOI:** 10.3390/s22020700

**Published:** 2022-01-17

**Authors:** Rana Alaa El-deen Ahmed, Manuel Fernández-Veiga, Mariam Gawich

**Affiliations:** 1 Business Information System, Arab Academy for Science Technology and Maritime Transport, Cairo B-2401, Egypt; 2 atlanTTic, Universidade de Vigo, 36310 Vigo, Spain; mveiga@det.uvigo.es; 3 Centre de Recherche Informatique, Université Française en Egypte (UFE), Cairo 1029, Egypt; mariam.gawish@ufe.edu.eg

**Keywords:** recommendation systems, neural collaborative filtering, ontologies, deep learning, retail dataset

## Abstract

Machine learning (ML) and especially deep learning (DL) with neural networks have demonstrated an amazing success in all sorts of AI problems, from computer vision to game playing, from natural language processing to speech and image recognition. In many ways, the approach of ML toward solving a class of problems is fundamentally different than the one followed in classical engineering, or with ontologies. While the latter rely on detailed domain knowledge and almost exhaustive search by means of static inference rules, ML adopts the view of collecting large datasets and processes this massive information through a generic learning algorithm that builds up tentative solutions. Combining the capabilities of ontology-based recommendation and ML-based techniques in a hybrid system is thus a natural and promising method to enhance semantic knowledge with statistical models. This merge could alleviate the burden of creating large, narrowly focused ontologies for complicated domains, by using probabilistic or generative models to enhance the predictions without attempting to provide a semantic support for them. In this paper, we present a novel hybrid recommendation system that blends a single architecture of classical knowledge-driven recommendations arising from a tailored ontology with recommendations generated by a data-driven approach, specifically with classifiers and a neural collaborative filtering. We show that bringing together these knowledge-driven and data-driven worlds provides some measurable improvement, enabling the transfer of semantic information to ML and, in the opposite direction, statistical knowledge to the ontology. Moreover, the novel proposed system enables the extraction of the reasoning recommendation results after updating the standard ontology with the new products and user behaviors, thus capturing the dynamic behavior of the environment of our interest.

## 1. Introduction

The amount of information found on web pages and social networks has increased dramatically in recent years as the Internet has grown. As a result, even while users have access to more information, it is becoming increasingly challenging to match their demands when it comes to providing information relevant to their interests. The rise of the Internet has also accelerated the spread of e-services across a variety of online platforms, with the primary benefit of providing products and services to consumers who have not yet purchased them anywhere and at any time. With so much data and services available, it is challenging not only for users to identify products they are interested in fast, but also for e-commerce and similar systems to recommend the product from the data. Recommendation systems (RSs) [1] are decision-support information systems created to assist users in locating things that fit their interests from the vast variety of choices [2,3].

There are three main sets of techniques for building personalized RSs: ontology-based [4,5], filtering by matrix factorization (MF) [6,7,8,9,10], and machine learning (ML) [11,12]. The common premise for all of them is trying to predict new items that match the users’ preferences, revealed through their past purchases or by means of explicit ratings. However, these approaches differ fundamentally: ontology-based systems use a formalized ontology (a conceptual graph of entities and their mutual relationships) suited to the specific domain for rule-based reasoning; MF attempts to discover the low-dimensional latent factors (hidden state variables) in the user–item preference matrix; ML estimates a statistical predictive model from the collected data. While these methods have shown effectiveness and accuracy in constructing RSs, all are based on a fixed ground truth (the dataset) captured at a given time instant, so it is difficult to recommend diversified, personalized products since the recommendations are based on past observed purchase history, and it is not possible to take advantage of the change in users’ preferences with time, their drift [13], because the computational load for building the model is often too large. For instance, ontologies have to follow a slow and complex synthesis process, and the aid of external experts is required [1]; ML needs large datasets and long training times to learn a precise statistical model, and MF runs in time $O(n^{3})$ at least, where *n* is the number of users. MF methods suffer also from the sparsity problem [14] (inaccurate inferences when new users or products are added), and ML methods exploit the statistical correlations independently of the logical relationships among user–items.

Though a hybrid RS (e.g., [15,16]) that combines two or more of the usual approaches seems intuitively more robust and able to tackles these problems, the drift of the users’ preferences with time cannot be tackled by hybrid RSs, yet, as long as the dataset used to build the system is not allowed to evolve. For this, a key property is that the changes in the dataset can be introduced automatically into an existing model and that the computational task of updating the model is low.

This paper presents a hybrid recommendation system (adapted for online retail markets) in which a dynamic ontology and a neural network classifier [17,18,19] work jointly to generate accurate recommendations for future purchases. The system differs from other works that attempt to incorporate semantics into the neural network (e.g., [20,21]) in that we allow the semantic representation to evolve with time, and in that, we use both item and user information to reveal the latent factor with the neural network. The main contributions of our work are:

- We propose a new ontology-based RS where the ontology is dynamically updated and evolved to capture the semantic relationships between users and products. In contrast to other knowledge-based systems, the evolution of the ontology is built automatically without the participation of experts;
- The novel proposed system enables the extraction of the reasoning recommendation results after updating the standard ontology with the new products and user behaviors;
- The proposed RS can be integrated seamlessly with other collaborative filtering and content-based filtering RSs;
- The proposed methodology is able to provide better recommendations, aligned with the current preferences of users.

The rest of the paper is organized as follows. Section 2 briefly introduces the tools and techniques used in our work, i.e., recommendation systems, neural collaborative filtering (NCF), and generalized matrix factorization (GMF). In Section 3, we review the related work. Section 4 gives an overview of the hybrid architecture of our proposed recommender and describes the main processing steps. The detailed architecture of the neural collaborative filtering component is presented next in Section 5, and Section 6 contains the experimental evaluation results of the system, including the description of the dataset, its preprocessing, the application of standard classification techniques, and their combination with the proposed neural network and the information provided by the dynamic ontology. Section 7 presents a separate evaluation of the personalized recommendations, and the paper makes some concluding remarks in Section 8.

## 2. Background

### 2.1. Recommending Systems

The two classical forms of creating RSs are collaborative filtering (CF) and content-based filtering (CBF). CF involves recommending products and items to active users who liked or purchased them in the past by comparing current user ratings (implicit or explicit) for things such as movies or products to those of similar users, which are nearest neighbors determined via some appropriate distance measure. This is used to provide recommendations for objects that have not yet been rated or seen by the active user. User-based and item-based approaches are the two most common types of this technique [14,22,23,24,25]. CBF promotes an item to users based on their interests and also the product description of their past purchase. Because of this, the main disadvantage of CBF is that the system’s recommended products are likely to be extremely similar to those that the active user has already purchased.

Generally, collaborative filtering involves matching the current user ratings for objects such as movies or products with those of similar users (nearest neighbors) to produce recommendations for objects not rated or seen by the active user. There are two basic variants of this approach, which are user-based and item-based collaborative filtering. Traditionally, within the previous category, the primary technique used to accomplish this task is the standard memory-based *K*-nearest-neighbor (KNN) classification approach. This approach compares a target user’s profile with the historical profiles of other users to find the top *K* users who have similar tastes or interests [26]. Other common forms of solving the CF problem include MF (with many variants [22,23,24,27]) and ML [11,12,28].

Content-based filtering RSs [9,12,29] promote an item to users based on their interests and the description of the item. Content-based recommendation systems could be used to propose web pages, news articles, restaurants, television shows, and objects for sale, among other things. Content-based recommendation systems share a method for: (i) describing the items that may be recommended; (ii) creating a user profile that describes the types of items the user likes; (iii) comparing items to the user profile to determine what to recommend, despite differences in the details of the domain systems [26].

### 2.2. Neural Collaborative Filtering

As highlighted above, the key step in CF is to find a good approximation to the user–item interaction function directly from the observed data. Latent matrix factorization postulates to this end a simple linear projection operator on a lower-dimensional space embedded in the feature space; yet, linear interactions might not be powerful enough to capture or replicate a complicated interaction function. An actual solution to this trap consists of replacing the linear latent factors by a more general representation for nonlinear user–item models. Currently, it is widely known that neural networks can faithfully learn any functional input–output relationship between observables and hidden variables [30], provided several mild technical conditions hold (basically, a bounded domain, an activation function under some general assumptions, and a growing number of nodes). The approximation is arbitrarily good if the width and depth of the neural network are unconstrained as well [31,32]. In other works, for a broad class of functions, deep neural networks are universal approximators, a very powerful result that can be established by first proving that neural networks can approximate well smooth and non-smooth simple functions (e.g., sawtooth functions and polynomials); then, these building blocks are used to approximate arbitrary functions by virtue of well-known results in functional analysis such as the Weierstrass approximation theorem or Lagrange’s interpolation. Further details were discussed at length in [32].

In view of this theoretical support, neural collaborative filtering, sparked by the recent influential paper [28], advocates quite naturally the use of a neural network in place of matrix factorization for modeling the interaction function f(·), under the intuition that the accuracy shown by neural network technology in an impressive array of machine learning tasks can also be exploited in CF. In NCF, the reduction in dimensionality is thus attained through a sequence of layers in a neural network [19], and the similarity among the latent factors is no longer restricted to be measured as a linear projection. More precisely, in NCF, the learned function can be written as the output of a neural network $\Phi_{\mathrm{NCF}}:R^{n_{0}}\to R^{n_{L}}$ of *L* layers:

(1) $$f\left(H,G\right)=\Phi_{\mathrm{NCF}}=\left\{\begin{array}{cc} W_{1}, & \;L=1\\ W_{2}\circ \sigma \circ W_{1}, & \;L=2\\ W_{L}\circ \sigma \circ W_{L-1}\circ \cdots \circ \sigma W_{1}, & \;L\geq 3 \end{array}\right.$$

where $W_{i}:R^{n_{i-1}}\to R^{n_{i}}$, $W_{i}\left(x\right):=A_{i}x+b_{i}$ are affine mappings giving the outputs of the *i*-th layer for inputs $x\in R^{n_{i-1}}$ coming out of the previous layer, ∘ denotes the composition of mappings, and σ(·) is a nonlinear activation function acting componentwise. $A_{i}\in R^{n_{i}\times n_{i-1}}$ and $b_{i}\in R^{n_{i}}$ are the parameters between layer i−1 and layer *i*. There is a plethora of activation functions proposed in the literature, but perhaps the most popular is still the rectified linear unit (ReLU) σ(x):=max(0,x). Note that $A_{i}$ is the connection or weight matrix connecting two consecutive layers of the neural network and that $b_{i}$ is the bias; these are known as the edge weights and node weights of the network, respectively. Note also that σ(x)−σ(−x)=x, σ(x)+σ(−x)=|x|, and σ(λx)=λσ(x) for all λ≥0; indeed, ReLU is no other thing than an ideal rectifier (a diode, in the language of electrical engineers).

Training in a neural network is performed by means of the optimization of a loss function through iterated backpropagation, i.e., the optimal edge and node weights $\left(A_{i},b_{i}\right)$ are computed backwards, from the output layer to the input layer. This is possible since the local gradient of the loss function with respect to the weights at layer *i* can be computed, so a general gradient descent sequence of steps can be followed. However, since the number of parameters in $\left(A_{i},b_{i}\right)$ can easily be too large, stochastic gradient descent (SGD) [33] substitutes the gradient by an empirical expectation calculated on the basis of a few training points (a batch), randomly sampled. The reduction in complexity with SGD is thus very substantial.

In practical applications, other numerical problems might arise: these are due to the unboundedness and non-differentiability of ReLU at zero, but both can be easily solved. For inferring probabilities, as is the case in CF, the typical squared loss function is better replaced by log-loss, namely the cross-entropy between the predicted output and the desired values.

### 2.3. Generalized Matrix Factorization

Since neural networks are universal approximators, NCF trivially generalizes matrix factorization as follows:

- One-hot encoding of users and items: The input to the NCF is a pair of unit vectors $e_{i}\in R^{\left|I\right|}$ and $e_{j}\in R^{\left|U\right|}$ (where I is the set of items, U is the set of users, and |·| denotes the number of elements of a set), which encode the identity of item i∈I and user j∈U, respectively. $e_{i}$ (resp., $e_{j}$) have a single one at coordinate *i* (resp., *j*), and their remaining elements are zero. These vectors are column-stacked $e_{i}⊙e_{j}:={\left[e_{i}^{T}\;e_{j}^{T}\right]}^{T}$, where ${}^{T}$ denotes the transpose of a vector, and are input to the network;
- The weight matrix is:
  (2) $$A_{1}=H⊗G,$$
  where ⊗ is the Kronecker product between two matrices, and the bias $b_{1}=0$. So $A_{1}\left(e_{i}⊙e_{j}\right)=g_{j}^{T}h_{i}$ for all i,j;
- The (output) activation function is the identity mapping, i.e., σ=I. Since matrix factorization is linear, a nonlinear activation function is completely unnecessary;
- The loss function is the mean-squared error (MSE).

These choices make the neural network $\Phi_{\mathrm{GMF}}$ discover the latent matrices G and H with the backpropagation algorithm and then act linearly on their inputs just multiplying the inner latent factors.

If, instead of using the identity activation function, one substitutes a nonlinear activation response ρ(·), while keeping the one-hot encoding and the internal Kronecker product, and uses as the loss function a proper $\ell_{p}$-norm, the result is an architecture that can operate as a *generalized matrix factorization* with a nonlinear response, encompassing many different types of decomposition.

### 2.4. Neural Matrix Factorization

Neural matrix factorization (NMF) is defined as the combination in a single recommendation system of GMF and NCF. Specifically, NMF builds a system that maps the input embeddings $e_{i},e_{j}$ to the outputs according to:

(3) $$\Phi \left(e_{i},e_{j}\right)=\Phi_{\mathrm{NCF}}\left(e_{i},e_{j}\right)+\Phi_{\mathrm{GMF}}\left(e_{i},e_{j}\right).$$

In [28], it was suggested that the input embeddings be split between the two parallel computation paths, that is,

(4) $$e_{i}=\left(e_{i1},\cdots ,e_{ip},e_{i(p+1)},\cdots ,e_{id}\right):=\left(e_{i,[1:p]},e_{i,[p+1:d]}\right),$$

and similarly for $e_{j}$, so that

(5) $$\Phi \left(e_{i},e_{j}\right)=\Phi_{\mathrm{NCF}}\left(e_{i,[1:p]},e_{j,[1:p]}\right)+\Phi_{\mathrm{GMF}}\left(e_{i,[p+1:d]},e_{j,[p+1:d]}\right);$$

yet, there is no loss of generality in using (3) always. Generalizations of (3) to other combination rules are straightforward, so, for instance, one could use $\Phi =\max\{\Phi_{\mathrm{NCF}},\Phi_{\mathrm{GMF}}\}$ if the outputs are probabilities, or a weighted average $\Phi =\pi_{0}\Phi_{\mathrm{NCF}}+\pi_{1}\Phi_{\mathrm{GMF}}$ for some prior $\left(\pi_{0},\pi_{1}\right)$. In the end, as in many other algorithms used in ML, (3) can be regarded as a form of ensemble averaging for only two different classifiers (recommenders, in our case). NMF is used in this paper for the fusion of ontology-based recommendation systems and neural network recommenders.

## 3. Related Work

Ontology-based recommenders are one sort of knowledge-based RS that has received attention when referring to better active user recommendations. An ontology—an organized set of concepts and categories in a subject matter or domain that formalizes their characteristics and relationships among them—can be used to combine diverse data and provide a first direction for recommendation preferences. Ontology models are used in an ontology-based RS for user profiling, personalized search, and Internet browsing [4,5] and support the expansion of RSs into a more diverse environment, allowing knowledge-based methods to be combined with traditional machine learning algorithms. Commercial RSs often include some simple product ontologies that can be used later via heuristics or a huge community of user’s actively rating content suited for collaborative recommendations. Both data and ontological background information can be represented in defined formats on the Semantic Web, where standard languages are used to represent metadata. Nonetheless, constructing an ontology-based RS is an expensive procedure that involves extensive knowledge expertise and handling of enormous datasets.

As for their use in RSs, ontologies have the advantage of an explicit modeling of semantic information from which logical inferences can be drawn using some standard rule-based inference system, such as Fact++ [34]. The system of concepts and qualitative relationships among them can be easily tracked and represented with graphical software tools such as Protégé editors (https://protege.stanford.edu, accessed on 30 November 2021) and several existing plugins for visualization and functional extension, one of these being the cellfie plugin used in this paper for the semi-automatic update of an ontology. Thus, ontologies enable a rich and detailed semantic modeling of information as a loosely structured system of intertwined concepts; yet, from a computational point of view, there exist some disadvantages in using them as the fundamental form of knowledge representation, which limits their usefulness in RSs. First, ontologies are highly specific to a domain realm, usually very narrowly focused. As such, building an ontology requires typically the participation of human experts for conceptualizing the main entities. In other words, building an ontology is far from being an automatic process; on the contrary, there is some risk of introducing bias due to the experts’ view of the field. Secondly, the ontology constitutes only an information base to which a set of complete inference rules are to be applied to deduce the implied consequences. Generally, these derived facts grow exponentially with the size of the ontology and quickly become unmanageable. Thirdly, building the ontology itself is costly in time and sometimes in access to experts. As a consequence, updating an ontology with new information frequently means rebuilding it from scratch, which is not realistic for a big RS such as on most websites or e-commerce sites.

In this paper, we used dynamic ontologies, i.e., ontologies that evolve with time, in a more general architecture for RSs, in combination with NCF. Our main purpose was to characterize the extent to which semantic modeling with ontologies and contemporary CF (namely, NCF) can complement each other by extracting at the same time statistical information and semantic knowledge from the same dataset in a semi-automatic way that overcomes most of the complexity involved in re-creating the ontology or re-training the NCF. In the rest of this section, we briefly review the related work on hybrid RSs and (neural) CF most related to our approach.

The work in [28] pioneered the research in merging neural networks and CF for novel RSs. The main idea, as mentioned previously, is to replace the linear matrix factorization commonly used in CF by a more general and potentially more effective function approximator: a (deep) neural network. The experiments reported in [28] showed a clear advantage when using this approach over the classical LMF that supports CF. A word of caution was introduced recently by [35], who repeated the experiments and found that, with a proper hyperparameter setting, LMF has similar or superior performance to NCF. In other words, the expressive power of neural networks appears not to be essential for modeling purposes. Therefore, the question is still open about the benefits and performance of using neural networks for inferring recommendations.

Despite the intriguing competitiveness, many other works have explored the use of deep networks for CF after [28]. For example, Reference [20] used two parallel neural networks joined in the last layers for learning item characteristics and user behavior together from text reviews. The first network works toward understanding users’ behavior from their reviews, while the second one understands the characteristics of the item from the reviews that are written on it. The last layer joins the two networks together, allowing latent factors learned for users and items to collaborate with each other in a similar way as with factorization techniques. Datasets named Yelp, Amazon, and Beer were used to test the algorithm, and the results of DeepCNNoutperformed all baseline RSs by an average of 8%. A comparison between neural collaborative filtering and matrix factorization was conducted in [35].

Reference [36] proposed an approach called neural semantic personalized ranking (NSPR) that combines the effectiveness of deep neural networks and pairwise learning. The semantic representation of the items that were combined with latent factors learned from implicit feedback was performed by NSPR to address the item cold-start recommendation task, specifically. Their system introduces two alternatives based on logistic and probit functions. The experiment of the proposed approach was performed on two datasets (Netflix and CiteuLike) and applied MF and topic-regression-based CF. The experiment proved that NSPR expressively outperformed the state-of-the-art baselines. The idea proposed by [20] for their innovative context-aware recommendation model was to use a convolutional matrix factorization (ConvMF) that incorporates convolutional neural networks into probabilistic matrix factorization. This had a clear effect on the sparsity problem. Again, the proposed model after integrating CNN into MF under a probabilistic perspective was able to improve the accuracy of the rating prediction in addition to capturing the contextual information of documents.

An example of a hybrid system composed of ontologies and CF is [33], applied to MOOCs. Their RS combines item- and user-based CF with an ontology to recommend for online learners personalized MOOCs within MOOC platforms. Here, the ontology was used in order to present a semantic explanation about the learner and MOOC that would be fused in the 11 recommendation method that would help enhance the personalization recommendation for the learner. The cold-start problem of the RS can be released by the use of the proposed hybrid technique by using the ontological knowledge before the initial data.

The calculation of the similarity between ontologies has also been addressed via machine learning techniques, as in [37,38]. The approach in this case was to perform a direct embedding of the graph ontology to simplify the detection of the similarity between the graphs and, next, to use the embedding as the input to a statistical learning algorithm. The main problem with those embeddings is the large size of the base ontology graphs, which for our application domain prevent the use of this sort of mapping.

## 4. Overview of the Proposed Recommendation System Architecture Based on ML, NCF, and Ontology Evolution

**Figure 1:**
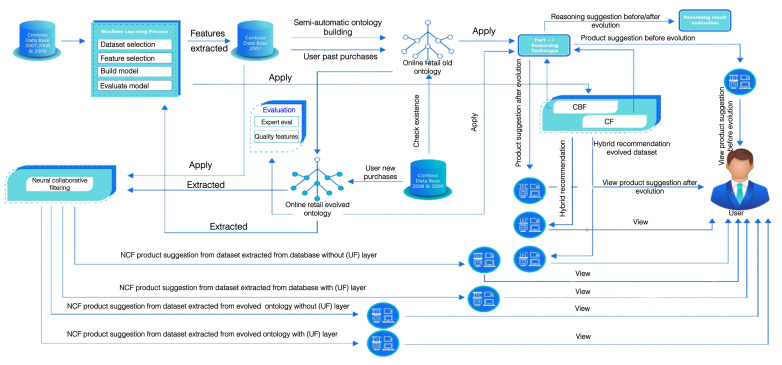
System architecture: Phase $\varphi_{1}$ is a pre-classification used for feature extraction (**top left**, machine learning process); Phase $\varphi_{2}$ uses the classification results and the features to build the base retail ontology (**middle** and **top right** blocks); Phase $\varphi_{3}$ derives the evolved ontology after adding the new information (**center** part); Phase $\varphi_{4}$ uses NCF (**middle left** block) jointly with CF and CBF (**top right**) to build the ensembled recommendations.

This section introduces the proposed recommendation system architecture based on machine learning, neural collaborative filtering, and ontology evolution, as well as the proposed neural collaborative filtering with ontologies framework based on GMF and NCF. The proposed system architecture is depicted in Figure 1 [5] and comprises four phases. Phase 1 $\varphi_{1}$ (top left of the figure) is the online retail dataset’s machine learning process; Phase 2 $\varphi_{2}$ (top middle) is the pre-evolution ontology of the online retail dataset; Phase 3 $\varphi_{3}$ (center part of the Figure) is the ontology after the evolution of the online retail dataset; finally, Phase 4 $\varphi_{4}$ (top right) is the neural collaborative filtering. The arrows represent the flow of information processing among the different computation steps:

- Phase $\varphi_{1}$ of the ML process starts by loading the online retail dataset for a three-year transaction and consulting a domain expert for the feature selection within the dataset. The feature selection is further complemented in Phase 2 with ML techniques, thus without subjective criteria. Along with that, the dataset is preprocessed and cleaned by removing noisy data or missing values. The dataset is then used for training, and the classification algorithm is built for the online retail domain. After that, the model is evaluated by calculating the accuracy, and the ML-based product suggestions are presented to the user after applying the hybrid recommendation techniques based on CF and CBF;
- Phase $\varphi_{2}$ includes the building of the online retail ontology before the evolution. The features selected in the machine learning process that give high accuracy are used as new inputs for enriching the old online retail ontology, which is built in a semi-automatic way with the standard cellfie plugin from the old dataset. This dataset records the users’ past purchases and behavior. The Fast Classification of Terminologies (Fact++) [34] reasoning plugin is applied to the old online retail ontology (before the evolution), which recommends the products for users depending on their similar characteristics, preferences, and past transactions by applying CF and CBF implicitly;
- Phase $\varphi_{3}$ entails the evolution of the old online retail ontology by using the 2008 and 2009 versions of the database; this evolution process takes place by checking both the old online retail ontology and the 2008 and 2009 database, then adding the new individuals to the old online retail ontology. As a result of this, the evolved online retail ontology is executed. The Fact++ reasoning plugin is applied to the evolved online retail ontology as in Phase 2, so new products suggestions will be shown to users according to the new purchases and behaviors. The two recommendations (before and after the evolution) are then compared to highlight the changes in the recommendations. Experimental results and examples are shown in Section 6. Afterwards, the evolved online retail ontology is extracted to apply to it the ML algorithms and obtain product suggestions to the user using hybrid recommendation techniques;
- Phase $\varphi_{4}$ applies NCF to the dataset extracted from the database, and recommendations are generated for the user both before and after adding the user feature layer (UF). The last step is the evaluation step. In order to execute the evaluation of the evolved ontology, two methods are used. The first one is the calculation of the precision and recall by a domain expert; the second method is implementing the quality features dimension by calculating the cohesion and conceptualization. Subsequently, the reasoning results of the old and the evolved online retail ontology are re-evaluated by the domain expert by calculating the precision and recall.

**Figure 2:**
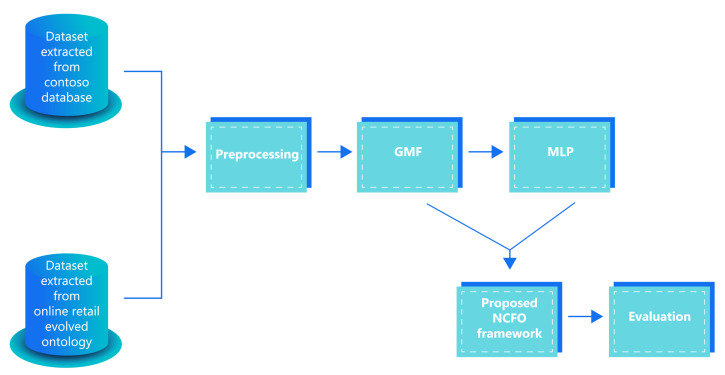
NCFO framework: the original and evolved databases are preprocessed first to encode the features. These are later input to a generalized matrix factorization and a neural collaborative filter to obtain the recommendations. The evaluation block measures the performance of the whole system.

Figure 2 presents the implementation steps that the proposed (NCFO) framework follows. The proposed NCFO framework includes five steps: the first section in the framework includes the two datasets used in the experiment; the first one is extracted from Contoso database, and the second dataset is extracted from the online retail evolved ontology. Then, the framework starts with its steps. First the preprocessing process is performed on both datasets. Second, the GMF method is used to formulate the proposed NCFO framework by applying the dot product between the MF user id and MF product id embedding vectors in addition to the MLP, which also uses the MLP user id and MLP product id embedding vector with the new layer of the user feature layer (UF) as the inputs for the MLP presented in the third step. Then, the three paths are concatenated with each other to form the proposed NCFO framework in the fourth step. Lastly, the fifth step is the evaluation, which occurs on both datasets before adding the user feature layer and after adding it.

## 5. Neural Collaborative Filtering Framework with Ontologies

**Figure 3:**
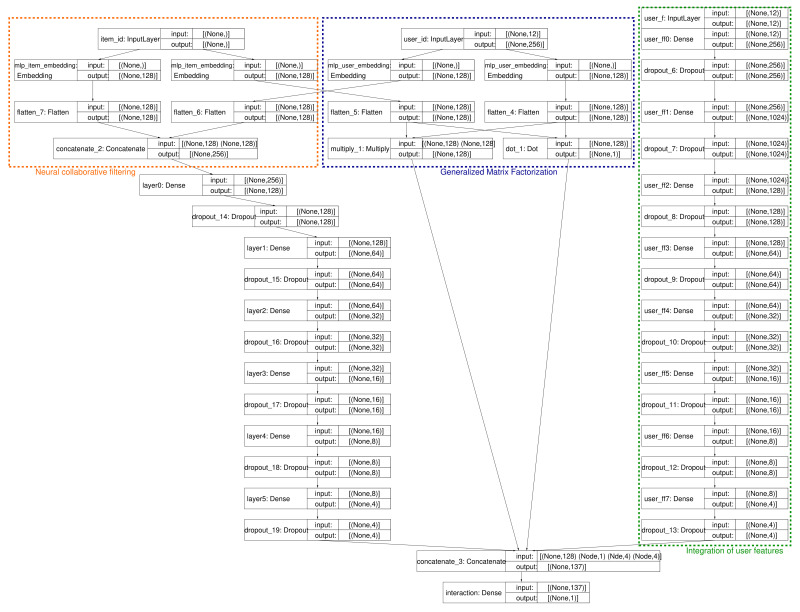
Full architecture and components of the proposed NMF hybrid classifier, part of Phase $\varphi_{4}$. There are three parallel processing paths, neural collaborative filtering (orange), generalized matrix factorization (blue), and the ontology embedding deep network (green).

The proposed NCFO framework for our retail market recommender is composed of the ensemble union of GMF and NCF, that is generalized matrix factorization and collaborative filtering, with a neural network as the function approximator. This is the main computation in Phase $\varphi_{4}$. The GMF block in the proposed architecture receives as the input the item and user embeddings (their ids, in simple one-hot encoding); in a parallel branch of computation, the NCF block takes as the inputs both the user and item embeddings, and the subset of features for the user id (user features (UFs)). The complete system architecture is depicted in Figure 3, which shows three internal blocks: the NCF part $\Phi_{\mathrm{NCF}}\left(\cdot \right)$ (bottom part in orange), the GMF part $\Phi_{\mathrm{GMF}}\left(\cdot \right)$ (middle section, in blue), and the novel deep neural network for integrating the ontology part (top section, in green), encoded as in (4). Each block shows its component layer, the input/output sizes, and the type of layer.

As seen in the figure, the UF is converted at the input layer to a sparse representation with one-hot encoding, before undergoing the remaining steps. Next, all the embedded input vectors (user, item, and UF) are processed by several layers of a neural network. Note that one branch of this union network implements the generalized matrix factorization approach, while the complementary branch works on the UF by applying a conventional neural network sequence of layers with a decreasing number of nodes in each layer. This second path of computation has naturally more layers than the GMF counterpart, as expected, since the UF encloses more richness than the latent factor modeling upon which the GMF works. The interaction between the two paths of prediction/classification happens mainly in the last (output) layer, where the inner representations found by each component are merged (by concatenation) and passed to the last hidden layer.

For the user and item embeddings, the dense neural layers have a decreasing number of units, in powers of two {128,64,32,16,8,4} and use ReLU as the activation function. The user features follow a deeper neural network with dense layers having {256,1024,128,64,32,16,8,4} units in each layer, also with ReLU. Note that, in both cases (except the expansion layer for the UF), the dropout factor was set to 0.5 for the connections between two consecutive layers. The activation function for the last (output) layer is sigmoid, and the loss function chosen was the binary cross-entropy, optimized via the Adam algorithm with a learning rate of 0.001. For training, the batch sizes were {32,64,128}, and the number of epochs was set to {100,200,400}. Training was performed for only a subset of the users, as the dataset was large, and testing with the Adam, Adagrad, and RMSprop optimizers.

## 6. Experimental Results

We now report on the implementation of the proposed algorithm and the experimental results obtained with the dataset. First, the preprocessing step to embed the classification techniques into the hybrid recommendation system is explained, so that this can be later used in the CF and CBF recommendations. Next, we present the implementation of the proposed novel neural collaborative filtering framework with ontology integration (NCFO). This proposal extends the recently developed neural collaborative framework [28], which already mixes CF and neural networks, with the information modeled with the ontology. We describe the modifications and advantages over the basic NCF approach and give an experimental performance evaluation. All the results were obtained running the experiments on a computer with an Intel(R) core i7 3.2 GHz processor with six cores and 16 GB RAM. The ML algorithms were implemented in Python 3.7 on the TensorFlow 2.0.0 and Keras 2.3.1 libraries. The tests for the ontology-based evaluation were performed on the same computer with the standard-purpose software Protègè and its complements, as described above.

### 6.1. Implementation Process Overview

**Figure 4:**
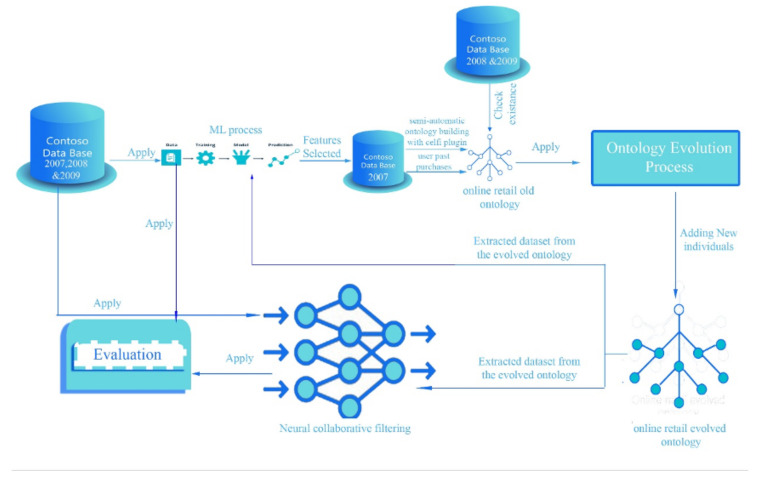
Detail of the integration of ontology evolution, ML, and NCF. This shows that the evolved ontology is one of the inputs for NCF.

Figure 4, which is itself a part of Figure 1, explains in detail the system implemented in this work for composing the hybrid RS. The system was composed of three parts; the input for the first part was the database for the years 2007, 2008, and 2009 on which the ML process was applied, which included feature selection and data cleansing, selecting the classification algorithms, model evaluation, and the visualization of results. The outputs were the selected features used in building the old online retail ontology, this one consisting of 60 classes, over 100,000 declaration axioms and more than 1.5M logical axioms from 113,953 individuals. The second part included the evolved online retail ontology after adding new individuals to the old ontology for the years 2008 and 2009. Next, the third part gave the evolved datasets that were extracted from the evolved online retail ontology to be the input for the ML and the NCF blocks.

### 6.2. Description of the Dataset

The datasets used in this experiment were two versions of the same primitive dataset. The first version was the original data (Contoso [39]), which included users’ features and the properties of products useful and typical for personalized user recommendations, such as customer unique tags, gender, economic and social status, geographic (i.e., cultural) information, etc. The second dataset, in turn, was obtained from the first after evolving an ontology built up from the raw data. Algorithm 1 summarizes the steps carried out for establishing the baseline classification results used during the integration.
**Algorithm 1:** Processing steps for data analysis (Phase $\varphi_{1}$)**Input**: datasets $D_{\mathrm{original}}$ and $D_{\mathrm{evolved}}$
Data curation, preparation, and cleansingFeature selection/extraction: expert + PCAGenerate the utility matrix *U* (user, item) interactionsGenerate the normalized sparsity matrix *S* (user, item) interactionsSVD decomposition of *S*: [U,Σ]←SVD(S) and truncation
Calculate similarity of latent factorsUnsupervised classification m∈{KNN,DT}(Accuracy,Precision,Recall,F1)←Classify(m,U·S)
Test and validate over $D_{\mathrm{original}}$ and $D_{\mathrm{evolved}}$



### 6.3. Feature Selection

**Figure 5:**
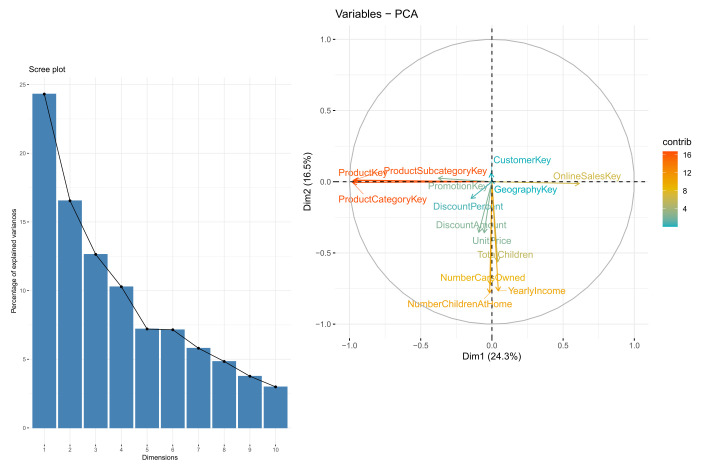
Principal component analysis of the dataset. The left panel shows the 10 first eigenvalues in decreasing order of the explained variance. The right panel depicts the correlations among features for the two highest principal components.

Let us first describe the essential information available in our database. The online retail dataset consisted of 36 features and 2,832,193 rows, for a total file size of over 800 MB. The number of unique customers was 18,484, while the number of unique products was 1860. Most of the features included in the dataset are self-explanatory (Table 1), and some of them are not significant for the recommendation outcomes. Among the numeric values, only Weight contained missing values for some of the entries (703,803 ≈24.85%), which were subsequently removed. For the experimental part, we determined that 10 features enclosed most of the necessary information using the two methods: advice from an external consultant and a PCA analysis of the raw data. Figure 5 shows the eigenvalues and percentages of variance explained by each of the numeric features. We see directly that the features were almost orthogonal along the two main principal components and that there was a substantial difference and correlation among the features. The cumulative variance explained by the 10 most significant eigenvalues was 95.83%. Based on this exploratory computation, we decided to keep 10 out of the 36 features of the dataset for the subsequent stages of the analysis, combining the latter results with the suggestions of an external expert in the area of retail markets. Feature extraction is part of Phase $\varphi_{1}$.

### 6.4. Unsupervised Classification with Ontology Integration

In order to quantify the benefits of integrating machine learning with other recommendation approaches—namely, ontology-based (OB) using formal logic for reasoning; CF or CBF, these being purely computational—we needed first to determine to what extent classical ML techniques can group and recognize as similar the user and item behaviors contained in the database. Since the dataset contained no labels as to the classes or profiles that the customers belonged, we were dealing with a typical unsupervised learning task. As a matter of fact, those classes or profiles were totally undefined in our setting, and the main goal of the ML task was then to implicitly define the features that could help to structure the data into disjoint subsets. Unsupervised learning is often characterized by the presence of latent or hidden variables that cannot be directly observed and arise only through noisy transformations in the raw data. In the following sections, we give the technical details for the processing steps outlined in Algorithm 2, which is the core of Phase $\varphi_{4}$.
**Algorithm 2:** Processing steps for data analysis, NCFO.**Input**: datasets $D_{\mathrm{original}}$ and $D_{\mathrm{evolved}}$Data curation, preparation, and cleansing**filter features**discard non-informative features: OnlineSalesKey, DiscountAmount,FirstName,LastName, ProductSubcategoryKey, ProductSubcategoryName, ProductCategoryKey, ProductCategoryName, UnitPrice, ClassName, BrandName, DateKey, BirthDate, Weight, DiscountPercent, PromotionType, StartDate, EndDate, AsiaSeason, EuropeSeason, IsWorkDay, PromotionKeyone-hot encoding: categorical features Gender, Education, MaritalStatus, CityName, StateProvinceName,RegionCountryNamereduce: group data by CustomerKey, ProductKeynormalize: linear normalization in [0,1]drop duplicatesUser-item embedding for generalized matrix factorization User-item embedding for neural matrix factorization
**one-hot encoding of users** $\mathrm{UserEmbedding}\leftarrow e_{1(i=\mathrm{user}-\mathrm{id})}$**one-hot encoding of items** $\mathrm{ItemEmbedding}\leftarrow e_{1(i=\mathrm{item}-\mathrm{id})}$**one-hot-encoding of user features** $\mathrm{FeatureEmbedding}\leftarrow e_{1(i=\mathrm{feature}-\mathrm{id})}$Ensemble classification of GMF, NMF, and NCF: training Evaluation over $D_{\mathrm{original}}$ and $D_{\mathrm{evolved}}$


### 6.5. Baseline Hybrid Classification

We repeated the same baseline experiments over the evolved ontology. To that end, the procedure consisted of evolving the original ontology, the one developed for the oldest version of the dataset. The evolved ontology gave rise to new predictions corresponding to the newly added items, and these new predictions were inserted back into the dataset as the ground truth. Then, the classifiers were applied again over the modified (evolved) dataset. The results appear in Table 2.

As shown in Table 2, there was an enhancement in the classification algorithms results after the evolution of the dataset. For KNN, the Accuracy increased substantially from 87% to 94% in the dataset created from the evolved retail ontology, while for decision trees (DTs), the accuracy rose from 73% to 87%. which is even more remarkable. In a similar fashion, Precision increased as well in both cases—KNN and DT— between the prior and posterior versions of the ontologies (datasets). The main conclusion to draw from these results is clear: the semantic relationship and recommendations found by the ontology, either in its static version or in its evolved offspring, when introduced back into the dataset, enriched the patterns and could be used to better train standard classification methods used in ML. We recall here that the main purpose of this numerical analysis was not to devise a good multi-class classifier for the retail data, but only to test whether the semantic information created by means of the ontology can be recognized and exploited by classical ML algorithms. The fact that some improvement in the performance can be measured ratifies the fact that the recommendations discovered via semantic rules contained fresh information not present in the original (not evolved) dataset. Consequently, the combination of ontology-based output and ML-based classifiers’ input was beneficial for inference and prediction, as intuitively expected. This step is part of Phase $\varphi_{3}$.

### 6.6. Neural Collaborative Filtering

In Phase $\varphi_{4}$, the NCF and the ontology information were blended to generate improved recommendations.

#### 6.6.1. Hyperparameter Setting

Based on our starting test cases, we decided to set a fixed split test threshold, namely 70%–30%, to strike a proper balance between overfitting and generalization. Therefore, this fraction was held constant over all the numerical experiments. Regarding the choice and tuning of the optimizer, we conducted tests with three optimizers, {Adam,Adagrad,RMSprop}, three batch sizes for evaluating the gradients, {32,64,128}, and different input sizes for training, as well as for testing, {100,200,400} different users. Numerical tests with more users are extremely intensive in computing time and were not attempted. Nevertheless, as we reported below, these combinations suffice to make effective predictions and recommendations, so we conjectured that little improvement is to be gained by using large input sizes. Naturally, this will depend on the diversity of the dataset.

**Figure 6:**
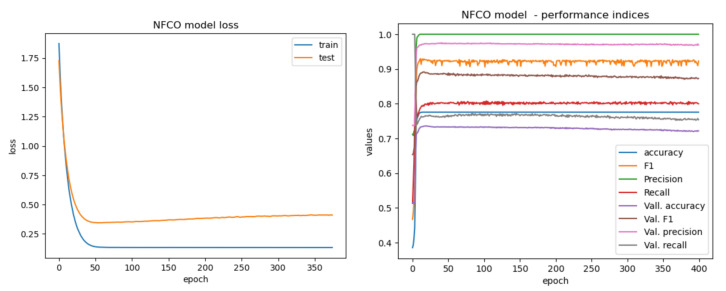
Convergence of the NCFO performance metrics.

Table 3 presents a summary of the results obtained for determining the setting of the optimizer parameters, as well as for assessing the performance achievable with the proposed NCFO architecture. These data correspond to the non-evolved dataset (i.e., the one before the evolution), since the behavior of the evolved dataset was exactly the same. Based on the results listed in Table 3, we selected the Adam optimizer (learning rate 0.001 with batch size 64 or 128 and 200 epochs for training). Longer training is more prone to cause the overfitting of the produced model, and as Table 3 reveals, there were no further consistent improvements by extending the training epochs beyond that value: convergence was attained well before that limit. Figure 6 shows the typical training and test loss curves, illustrating the convergence of the system around Epoch 100, consistently for every one of the performance metrics.

#### 6.6.2. Running Time

The running time of the training step for the NCFO hybrid system depended crucially on the number of internal parameters in the neural networks, the number of epochs, and the number of the training samples. To a lesser extent, the computing time for training the system was weakly dependent on the optimizer chosen. Though a complete analytical characterization of the computational complexity for our system was not possible—the internal optimization procedure via backpropagation is stochastic—we list in Table 4 some representative results for our tests. We concluded from these figures that the optimizer RMSprop required more than twice the training time and that the training time decreased almost linearly with the batch size and increased linearly with the number of users used for the training dataset. In view of these tests, we decided to take batch sizes of 64 and 128 for the evaluation and 200 epochs for training the algorithm, since these attained a good balance between the model accuracy and loss and the total running time.

#### 6.6.3. Performance

Next, we characterize the performance of the NCFO framework when making recommendations. To this end, we first present the performance results of the hybrid neural architecture when the input was the original non-evolved database and after the ontology evolution. Thus, using the hyperparameters for the configuration selected according to the criteria in the two latter subsections (recall, Adam optimizer with learning rate 0.001, batch size equal to 64 and 128, and 200 epochs for training), we obtained the results listed in Table 5.

Table 5 contains the measured validation accuracy and validation loss for different test cases, as a function of the training dataset size, and compares the performance when the input dataset came from the non-evolved ontology (i.e., the original old dataset) and when the ontology was evolved. Furthermore, we compare in the same table the results when the NCFO architecture disregarded the user features (No UF) and when those user features were included (UF). The former case represents the NCFO architecture without considering the information provided by the ontology, evolved or not. In other words, this is the equivalent system to the generalized matrix factorization (GMF) implemented through the neural collaborative filtering approach and should be compared to a standard CF recommender eventually. The latter case corresponds to the NCFO architecture with the complementary information given by the (evolved or non-evolved) ontology, or the full architecture previously depicted in Figure 3.

**Figure 7:**
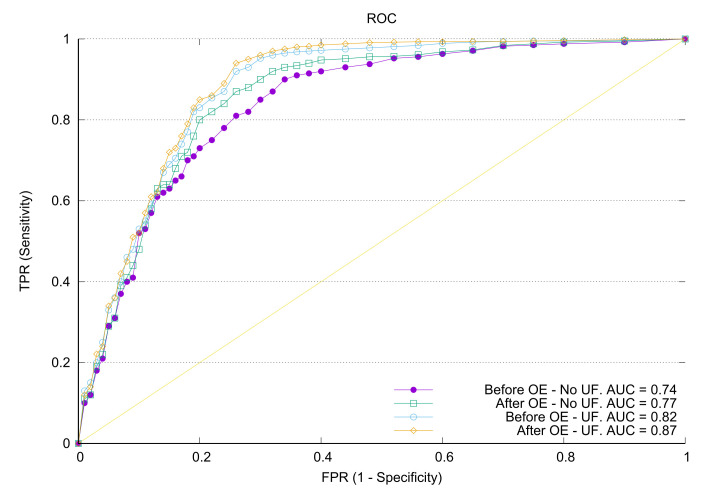
ROC for the different versions of the dataset: without ontology evolution and without user features. The curves are plotted for Multiclass 1 vs. all classifications.

As we can see in Table 5, there was a measurable improvement along both axis, i.e., when the user features output by the ontology reasoner were taken into account, and also when the novel information preprocessed by the ontology was used to train the system. Therefore, these experimental results confirmed our original intuition that implicit information contained in a domain-specific ontology can be exploited in a generic ML architecture. Moreover, we can also verify that training the system with increasingly large numbers of users and items also was useful to improve the results, since it increased the variety of patterns to which the NCFO neural network was exposed. Indeed, if the input dataset had enough variance in the raw data, using more training cases did not lead automatically to overfitting of the model. This latter assertion, however, needs to be carefully verified, since it is very sensitive to the (lack of) similarity in the available dataset. This study has been left out of the scope of the paper, nevertheless. The receiver operations characteristic (ROC) for the NCFO classifier is plotted in Figure 7. It was calculated using the one vs. rest policy (our classifiers were multiclass), and it confirmed the gains attained by incorporating the evolution and UFs into the hybrid RS.

The second methodology for evaluation was the calculation of the hit ratio with the proposed hybrid recommender. Recall that the recommender outputs a list of items suggested for a given user, the top *k* best items according to the algorithm that best suit her/his preferences, where *k* is a configuration parameter (k=1,3,5,10, for instance). Namely, the proposed NCFO classification system ranks the items in decreasing order of relevance according to the output of the ensemble neural classifier and selects the *k*-highest-ranked products for recommendation, where *k* can be set as a parameter by the user. For the calculation of the hit ratio, we followed the common *leave-t-out* approach [17] followed in most of the literature. We selected randomly and uniformly a subset L of users from the test set. For each user u∈L, we picked her/his *t* most relevant items $i_{1},\cdots ,i_{t}$ from the utility matrix and removed them as if there had not been any interactions $\left(u,i_{j}\right)$ in the test dataset. Next, we predicted the top k≥t items for user *u* with the NCFO approach and counted a hit (count one) if item $i_{j}$ was one of the items in the recommended list R for some j=1,⋯,t and a miss (count zero) otherwise. The size of the list L depended in our case on the size of the test dataset, which was in turn 30% of the total dataset, where this ranged from 100 to 400 users. In our case, we set t=k for all values k=1,5,10, i.e., for top-1, top-3, and top-5 recommendation lists.

Note that the definition of the top-*k* hit ratio is stringent for k=1, as we required strictly that the recommended item be exactly the one suppressed from the test dataset. In contrast, the requirement for the hit ratio became more loose as *k* increased, since we required only that at least one of the items in R coincide with the best-ranked items by user *u*, or $R\cap \{i_{1},\cdots ,i_{t}\}\neq \emptyset$. Moreover, note that the special case k=0 corresponds to the case where the recommendation list R is disjoint with the best-ranked list $\left\{i_{1},\cdots ,i_{t}\right\}$, so the user obtains as recommended products a list of novel or different ones than the one she/he already purchased and knows.

The results of the experiment for the hit ratio are presented in Table 6. We can see that the hit ratio for the top-one recommendation was rather low, since the system had to correctly identify the unique top-ranked product for the user. There was, however, a slight improvement when the user features and the ontology evolution were individually incorporated into the system. The performance improved substantially when we increased the value of *k* and enlarged the list of recommended products. In this way, for k=3, we obtained an average hit ratio around 65%, and again, we saw an improvement when the ontology evolution was considered, as well as when the user features were taken into account. In a similar way, the performance still improved up to around a 77% hit ratio if the list was expanded to the top-five recommendations for the random users. The key aspect to note is that the inclusion of the user features and the ontology evolution information led consistently to higher hit ratios, in the range of 3–4%. While this margin is moderate, it is meaningful, since we did not put special effort into designing an optimal neural network architecture. Accordingly, we conjectured that with more fine-tuning and optimized layers and dimensions, the achievable hit ratio can still be increased.

## 7. Online Retail Personalized Recommendations

### 7.1. Recommendation Results Based on Ontology Reasoning

The Fact++ reasoning plugin was applied on the online retail ontology. Each customer individual in the ontology had data property assertions such as age, gender, number of children, material status, education, and the order details for each individual. The reasoner detects the similarities between the customer individuals and recommends products semantically according to these similarities. The results are collected in Table 7.

To simplify the recommendation results, we randomly selected two pairs of users (*u*_1_; *u*_2_) and (*v*_1_; *v*_2_) who were very similar to each other according to the cosine similarity measure (of their latent factors). Specifically, their similarities were 0.997 and 0.982.

After the evolution, new products were added to the ontology, and the customers bought the new products that were added after the evolution. Therefore, the reasoner after the evolution recommended from the new products that were added according to the change of their behaviors. After the evolution, new products (individuals) were added to the ontology. According to that, all the test cases bought new products that were added after the evolution. Therefore, the reasoner after the evolution recommended from the new products that were added according to the change of their behaviors.

### 7.2. Recommendation Results Based on ML and the NCFO

This section presents the personalized recommendations to customers according to their similarities, by using several techniques such as the machine learning recommendation by using the hybrid recommendations techniques from the initial dataset extracted from the database and the second dataset extracted from the evolved online retail ontology. After that, the recommendations extracted by the neural collaborative filtering using the deep learning techniques are presented as well, both before adding the user feature layer and after adding it, and for the two settings of recommendation before and after the evolution of the ontology.

The results included in this section constitute examples of recommendations for individual users in every case. We recall that the average quality of the recommendation was evaluated through the intrinsic performance of the NCFO (validation accuracy and loss) and additionally through the hit ratio, which measures the fraction of adequacy among the recommended products and those highly ranked by a sample of users. The hit ratio is meaningful in that we did not have the possibility of collecting the opinions of the users on the created recommendations, so feedback was not possible in our case.

Specifically, the lists of recommended products appearing in the tables below correspond to the case of the top-five recommendations obtained for the *leave-zero-out* policy. In other words, we did not remove from the test dataset any of the top-five highly ranked products (HRPs) already purchased by that user. As a result, the recommended products were disjoint with the HRPs. We emphasize that, in some test cases, the HRPs can be a multiset, i.e., the same product can appear more than once if there are not enough explicit preferences declared by that user according to her/his purchase history.

To simplify the recommendation results, we randomly selected two pairs of users $\left(u_{1},u_{2}\right)$ and $v_{1},v_{2})$ who were very similar to each other according to the cosine similarity measure (of their latent factors). Specifically, their similarities were 0.997 and 0.982. Consequently, the purpose of the following examples is to give an empirical sample of the consistency or coherence of the recommendations. Formally, one possibility for measuring the coherence is to use the Jaccard distance:

(6) $$J\left(A,B\right)=\frac{\left|A\cap B\right|}{\left|A\cup B\right|}$$

between the respective lists A, B of their recommended products, i.e., the fraction of overlap between the two lists (J(A,A)=1, J(A,B)=J(B,A)). A compound measure of coherence for a given set of test users U could then be defined as:

(7) $$C=\frac{1}{\left|U|\cdot (|U|-1\right)}\sum_{u,v\in U,u\neq v}d\left(u,v\right)J\left(R_{u},R_{v}\right),$$

just by normalizing the aggregate pairwise coherence, where d(u,v) is the cosine similarity between u,v and $R_{u}$ (resp., $R_{v}$) denotes the set of items recommended to users *u* (resp., *v*). However, the coherence Formula (7), despite its simplicity, does not lend itself to a clear intuitive interpretation. The reason is that it aggregates the individual coherence according to the cosine similarities, so it depends functionally in a non-trivial way on the probability density function of d(u,v). Conversely, two very different distributions can have close values of the coherence (7). Since comparing two distributions—directly for d(u,v) or for the transformed coherence—can be performed in several forms depending on the statistical applications, we decided not to work with the aggregated measure and simply show several test cases to give a rough idea of the practical results.

In Table 8, Table 9 and Table 10, we present the outcomes of the recommendations for the first hybrid system (ontology + classification with KNN and DT) and the highly ranked products (HRPs) and recommendation lists for the neural NCFO hybrid system. In every case, the results with and without the inclusion of the user features are included, and for the NCFO, the results before and after the evolution of the ontology are given as well. This allows a direct comparison between the textual similarities of the recommendation lists. We can easily check that, for the two pairs of users who were strongly similar to each other (in cosine distance), the recommendation lists overlapped significantly, as expected. As explained above, this is a simple form of verification of the coherence of the recommendations.

### 7.3. Evaluation of the Results

The reasoning recommendation results of the online retail ontology before the evolution were presented to the domain expert to evaluate the recommendations generated for the users that were used in the experiment. The expert identified 17 correct recommendations, and the total number of all recommendations was 27. Then, the precision was:

(8) $$\mathrm{Precision}=\frac{\mathrm{number}\;\mathrm{of}\;\mathrm{correct}\;\mathrm{recommendations}}{\mathrm{total}\;\mathrm{number}\;\mathrm{of}\;\mathrm{recommendations}}\frac{17}{27}=63\%.$$

The expert also mentioned 15 recommendations that did not exist in the online retail ontology before the evolution. Then, the total number of possible recommendations equaled 32. According to this, the recall was:

(9) $$\mathrm{Recall}=\frac{\mathrm{number}\;\mathrm{of}\;\mathrm{correct}\;\mathrm{recommendations}}{\mathrm{total}\;\mathrm{number}\;\mathrm{of}\;\mathrm{possible}\;\mathrm{recommendations}}=53.12\%.$$

The reasoning recommendation results of the online retail ontology after the evolution were presented to the domain expert to evaluate the recommendations generated for the users that were used in the experiment. The expert identified 20 correct recommendations, and the total number of all recommendations was 23. Then, the precision Precision=20/23=86.95%. Finally, the expert’s evaluation reported 10 recommendations that did not exist in the online retail ontology before the evolution. Then, the total number of possible recommendations equaled 30. Then, the recall in this case $\mathrm{Recall}=\frac{20}{30}=67\%$. We therefore saw that the evolved ontology, even if increased with a small fraction of its original size, substantially improved over the original performance values.

## 8. Conclusions and Remarks

The results reported in this work showed evidence allowing us to draw two main conclusions:

- The information extracted by a logical reasoner based on a suitable ontology and in parallel from a neural collaborative filter can be combined so that the accuracy of the recommendations is improved. We showed results in this respect for the classification accuracy and also for the hit ratio, which is more meaningful for the recommendation of products;
- Another dimension that can effectively be exploited to improve the quality of predictions is the evolution of the ontology. Thus, a feedback loop in which novel data are inserted back again into the ontology provides a two-fold benefit: it allows the system to evolve in time, capturing the time-varying behavior of their preferences, if present; it combines naturally fresh information with past information without having to externally weigh the impact of each factor.

## Figures and Tables

**Table 1:** A summary of the features in the dataset.

Type	Features
Key (6)	OnlineSalesKey, CustomerKey, GeographicKey, ProductKey, ProductSubcategoryKey, ProductCategoryKey
Numeric (9)	DiscountAmount, TotalChildren, NumberCarsOwned, YearlyIncome, NumberChildenAtHome, UnitPrice, Weight, PromotionKey, DiscountPercent
String (17)	FirstName, LastName, Gender, Education, MaritalStatus, CityName, StateProvinceName, RegionCountryName, ProductName, ProductSubcategoryName, ProductCategoryName, ClassName, BrandName, PromotionType, AsiaSeason, EuropeSeason, IsWorkDay
Date (4)	BirthDate, StartDate, EndDate, DateKey

**Table 2:** Performance for the simple classifiers before and after the evolution of the ontology.

	Before Evolution		After Evolution	
	KNN	DT	KNN	DT
Accuracy	87.47%	73.29%	94.06%	87.61%
Precision	99.18%	99.22%	99.31%	99.11%
Recall	99.07%	86.68%	96.35%	92.40%
F1	98.62%	92.52%	97.80%	95.63%

**Table 3:** Soft accuracy for different configurations of the parameters. Dataset without ontology evolution.

			Optimizer		
(# of Users, # of Products)	# of Epochs	Batch Size	Adam	Adagrad	RMSprop
(50,304)	100	32	0.8649	0.7215	0.8522
		64	**0.8705**	0.7246	0.8548
		128	0.8667	0.7195	0.8523
	200	32	0.8847	0.7369	0.8673
		64	0.8834	0.7428	0.8702
		128	**0.8894**	0.7419	0.8726
	400	32	0.8980	0.7962	0.8644
		64	0.8910	0.8017	0.8784
		128	0.8934	0.8027	0.8732
(100,418)	100	32	0.8867	0.7547	0.8727
		64	0.8969	0.7568	0.8837
		128	0.8955	0.7611	0.8868
	200	32	0.8914	0.7725	0.8782
		64	0.8959	0.7842	0.8926
		128	0.8891	0.7844	0.8968
	400	32	0.8909	0.7753	0.8865
		64	0.9037	0.7856	0.9026
		128	0.9064	0.7839	0.9030
(200,553)	100	32	0.8894	0.8065	0.8676
		64	0.9026	0.8148	0.8828
		128	0.9063	0.78361	0.8912
	200	32	0.8946	0.8273	0.8964
		64	0.9131	0.8479	0.9028
		128	0.9092	0.8622	0.9003
	400	32	0.9128	0.8341	0.9074
		64	0.9107	0.8318	0.9056
		128	0.9116	0.8493	0.9061

**Table 4:** Training times for the NCFO architectures (seconds).

			Optimizer		
(# of Users, # of Products)	# of Epochs	Batch Size	Adam	Adagrad	RMSprop
(50,304)	100	32	145.59	189.61	141.35
		64	92.94	93.99	94.92
		128	62.35	63.99	63.37
	200	32	278.63	285.06	278.89
		64	184.44	190.17	181.16
		128	122.95	119.85	119.46
	400	32	566.35	555.67	565.06
		64	367.53	374.39	360.75
		128	240.70	236.37	236.96
(100,418)	100	32	250.34	249.61	568.37
		64	162.49	163.74	381.78
		128	109.51	106.37	245.87
	200	32	486.97	499.78	1158.23
		64	320.06	323.31	752.08
		128	211.67	205.29	483.98
	400	32	967.71	993.87	2750.17
		64	651.75	1518.80	2127.64
		128	403.53	949.84	397.96
(200,553)	100	32	482.37	484.87	—
		64	301.06	305.10	—
		128	198.08	189.73	—
	200	32	965.60	1213.02	—
		64	626.58	603.84	—
		128	402.97	389.71	—
	400	32	2005.65	1880.86	—
		64	1240.91	1177.66	—
		128	756.34	757.84	—

**Table 5:** Validation of the soft accuracy (acc.) and validation loss (loss) for the proposed NCFO hybrid recommendation architecture.

	Before OE				After OE			
Training	No UF		UF		No UF		UF	
(# of Users, # of Products)	Acc.	Loss	Acc.	Loss	Acc.	Loss	Acc.	Loss
(50,304)	87.33	0.3574	89.15	0.3777	92.43	0.3215	**94.58**	0.3186
(100,418)	88.23	0.3296	89.19	0.3408	92.81	0.3309	95.48	0.3325
(200,553)	88.33	0.2908	90.62	0.2993	93.54	0.3005	96.76	0.3016
(400,840)	88.94	0.2215	90.39	0.2344	93.25	0.2150	96.68	0.2192

**Table 6:** Hit ratio with NCFO. Impact of the training dataset size and the ontology evolution.

Top-1 recommendation				
Training	Before OE		After OE	
(# of Users, # of Products)	No UF	UF	No UF	UF
(50,304)	0.331	0.367	0.358	0.383
(100,418)	0.352	0.370	0.368	0.388
(200,553)	0.356	0.371	0.370	0.391
Training	Before OE		After OE	
(# of Users, # of Products)	No UF	UF	No UF	UF
(50,304)	0.624	0.651	0.648	0.662
(100,418)	0.646	0.657	0.661	0.674
(200,553)	0.648	0.659	0.661	0.676
**Top-5 recommendations**				
Training	Before OE		After OE	
(# of Users, # of Products)	No UF	UF	No UF	UF
(50,304)	0.733	0.749	0.752	0.778
(100,418)	0.765	0.763	0.778	0.797
(200,553)	0.770	0.774	0.783	0.807

**Table 7:** Ontology reasoning recommendation before and after ontology evolution.

$\left(u_{1},u_{2}\right)$—before evolution—$\left(v_{1},v_{2}\right)$				
RP	1. Contoso telephoto conversion lenx 400 silver	1. MGS Hand Games women M400 silver	1. Litware home theater system 5.1 channel M51 Black	1. Contoso telephoto conversion lensx400silver
	2. Adventure works 26,720 PLCDHTVM 140 silver	2. Adventure works 26,720 PLCDHTVM 140 silver	2. Adventure works 26,720 PLCDHTVM 140 silver	2. Adventure works 26,720 PLCDHTVM 140 silver
	3. sv16xDVDM 360 Black	3. sv16xDVDM 360 Black	3. sv16xDVDM 360 Black	3. sv16xDVDM 360 Black
	4. Contoso Home Theater system 5.1 channel M 1520 white	4. Contoso Home Theater system 5.1 channel M 1520 white	4. MGS Hand Games for office worker L299 silver	4. Contoso 4GMP3 player E400 silver
	5. Contoso 4G MP3 player E400 silver	5. MGS Hand Games for office worker L28 Black	5. SV Hand Games for office worker L28 Red	5. Contoso Home Theater system 5.1 channel M1520 white
		6. MGS Hand Games for office worker L299 Red	6. Contoso Home Theater system 4.1 channel M1410 Brown	
$\left(u_{1},u_{2}\right)$—**after evolution**—$\left(v_{1},v_{2}\right)$				
RP	1. Contoso telephoto Conversion lensX400 silver	1. MGS Hand Games women M400 silver	1. Litware Home theater system 5.1 Channel M515 Black	1. Contoso telephoto conversion Lens X400 Silver
	2. Contoso 4G MP3 player E400 silver	2. Contoso home theater system 5.1 channel M1520 white	2. SV Hand Games for office worker L28 Black	2. Contoso 4G MP3 player E400 silver
	3. Contoso home theater system 5.1 channel M1520 white		3. MGS Hand Games for office worker L299 Yellow	3. Contoso 4GMP3 player E400 Silver
		4. MGS Hand Games for office worker L299 Black	4. Contoso Home Theatre system 5.1 channel M1520 white	
			5. Contoso Home Theatre system 5.1 channel M1520 white	5. Contoso Home Theatre 4.1 channel M1410 Brown
			6. SV Hand Games for office worker L28 yellow	6. MGS Gears of war 2008 M450
			7. MGS Hand Games for office worker L299 Silver	7. MGS collector’s M160

**Table 8:** Top-five recommendation for two pairs of aligned users using the KNN and DT classifiers.

$\left(u_{1},u_{2}\right)$—before ontology evolution—$\left(v_{1},v_{2}\right)$				
KNN	1. SV 16xDVD M360 Black	1. SV 16xDVD M360 Black	1. Adventure Works 26" 720p LCD HDTV M140 Silver	1. Adventure Works 26" 720p LCD HDTV M140 Silver
	2. Contoso 512MB MP3 Player E51 Silver	2 Contoso 512MB MP3 Player E51 Silver	2. SV 16xDVD M360 Black	2. SV 16xDVD M360 Black
	3. Contoso 512MB MP3 Player E51 Blue	3. Contoso 512MB MP3 Player E51 Blue	3. A. Datum SLR Camera X137 Grey	3. A. Datum SLR Camera X137 Grey
	4. Contoso 1G MP3 Player E100 White	4. Contoso 1G MP3 Player E100 White	4. Contoso Telephoto Conversion Lens X400 Silver	4. Contoso Telephoto Conversion Lens X400 Silver
	5. Contoso 2G MP3 Player E200 Silver	5. Contoso 2G MP3 Player E200 Silver	5. Contoso Optical USB Mouse M45 White	5. Contoso Optical USB Mouse M45 White
DT	1. Fabrikam Refrigerator 24.7CuFt X9800 White	1. Fabrikam Refrigerator 24.7CuFt X9800 White	1. A. Datum SLR Camera X137 Grey	1. A. Datum SLR Camera X137 Grey
	2. Contoso 512MB MP3 Player E51 Silver	2. Contoso 512MB MP3 Player E51 Silver	2. Contoso Telephoto Conversion Lens X400 Silver	2. Contoso Telephoto Conversion Lens X400 Silver
	3. Contoso 512MB MP3 Player E51 Blue	3. Contoso 512MB MP3 Player E51 Blue	3. Contoso Optical USB Mouse M45 White	3. Contoso Optical USB Mouse M45 White
	4. Contoso 1G MP3 Player E100 White	4. Contoso 1G MP3 Player E100 White	4. SV Keyboard E90 White	4. SV Keyboard E90 White
	5. Contoso 2G MP3 Player E200 Silver	5. Contoso 2G MP3 Player E200 Silver	5. NT Bluetooth Stereo Headphones E52 Blue	5. NT Bluetooth Stereo Headphones E52 Blue
$\left(u_{1},u_{2}\right)$—**after ontology evolution**—$\left(v_{1},v_{2}\right)$				
KNN	1. SV Hand Games for Office worker L28 Red	1. SV Hand Games for Office worker L28 Red	1. A. Datum SLR Camera X137 Grey	1. A. Datum SLR Camera X137 Grey
	2. Contoso 2G MP3 Player E200 Silver	2. Contoso 2G MP3 Player E200 Silver	2. Contoso Telephoto Conversion Lens X400 Silver	2. Contoso Telephoto Conversion Lens X400 Silver
	3. Contoso 2G MP3 Player E200 Black	3. Contoso 2G MP3 Player E200 Black	3. Contoso Optical USB Mouse M45 White	3. Contoso Optical USB Mouse M45 White
	4. Contoso 4G MP3 Player E400 Silver	4. Contoso 4G MP3 Player E400 Silver	4. SV Keyboard E90 White	4. SV Keyboard E90 White
	5. Contoso 8GB Super-Slim MP3/Video Player M800	5. Contoso 8GB Super-Slim MP3/Video Player M800	5. Contoso 4G MP3 Player E400 Silver	5. Contoso 4G MP3 Player E400 Silver
DT	1. SV Hand Games for Office worker L28 Red	1. SV Hand Games for Office worker L28 Red	1. SV Keyboard E90 White	1. SV Keyboard E90 White
	2. Contoso 2G MP3 Player E200 Silver	2. Contoso 2G MP3 Player E200 Silver	2. Contoso 4G MP3 Player E400 Silver	2. Contoso 4G MP3 Player E400 Silver
	3. Contoso 2G MP3 Player E200 Black	3. Contoso 2G MP3 Player E200 Black	3. NT Bluetooth Stereo Headphones E52 Blue	3. NT Bluetooth Stereo Headphones E52 Blue
	4. Contoso 4G MP3 Player E400 Silver	4. Contoso 4G MP3 Player E400 Silver	4. SV 40GB USB2.0 Portable Hard Disk E400 Silver	4. SV 40GB USB2.0 Portable Hard Disk E400 Silver
	5. Contoso 8GB Super-Slim MP3/Video Player M800	5. Contoso 8GB Super-Slim MP3/Video Player M800	5. Contoso USB Cable M250 White	5. Contoso USB Cable M250 White

**Table 9:** Top-four recommendations (NCFO) and highly rated products for two pairs of aligned users. Original dataset.

$\left(u_{1},u_{2}\right)$—before user features—$\left(v_{1},v_{2}\right)$				
HRP	1. Contoso 4GB Portable MP3 Player M450 White	1. Litware Washer & Dryer 21in E214 Silver	1. NT Washer & Dryer 21in E2100 White	1. MGS Hand Games men M300 Black
	2. NT Washer & Dryer 21in E2100 White	2. MGS Age of Empires III: The Asian Dynasties M180	2. MGS Hand Games men M300 Black	2. Litware Washer & Dryer 21in E214 Silver
	3. Contoso 4GB Portable MP3 Player M450 White	3. MGS Age of Empires III: The Asian Dynasties M180	3. MGS Age of Empires III: The Asian Dynasties M180	3. MGS Age of Empires III: The Asian Dynasties M180
	4. Contoso 4GB Portable MP3 Player M450 White	4. MGS Age of Empires III: The Asian Dynasties M180	4. MGS Age of Empires III: The Asian Dynasties M180	4. MGS Age of Empires III: The Asian Dynasties M180
NCFO	1. Contoso USB Cable M250 Blue	1. NT Wireless Bluetooth Stereo Headphones M402 Green	1. MGS Dungeon Siege: Legends of Aranna M330	1. Contoso Washer & Dryer 25.5in M255 Green
	2. Contoso Washer & Dryer 25.5in M255 Green	2. Contoso 4GB Portable MP3 Player M450 Black	2. NT Wireless Bluetooth Stereo Headphones M402 Red	2. Contoso Digital camera accessory kit M200 Black
	3. Contoso 4GB Portable MP3 Player M450 Black	3. Litware Washer & Dryer 25.5in M350 Silver	3. Fabrikam Trendsetter 1/2${}^{\prime \prime }$ 3 mm X300 Black	3. NT Wireless Bluetooth Stereo Headphones M402 Green
	4. MGS Return of Arcade Anniversary Edition M390	4. NT Washer & Dryer 24in M2400 Green	4. MGS Flight Simulator 2000 M410	4. Contoso 4GB Portable MP3 Player M450 Black
$\left(u_{1},u_{2}\right)$—**after user features**—$\left(v_{1},v_{2}\right)$				
HRP	1. Contoso 4GB Portable MP3 Player M450 White	1. Litware Washer & Dryer 21in E214 Silver	1. NT Washer & Dryer 21in E2100 White	1. MGS Hand Games men M300 Black
	2. NT Washer & Dryer 21in E2100 White	2. MGS Age of Empires III: The Asian Dynasties M180	2. MGS Hand Games men M300 Black	2. Litware Washer & Dryer 21in E214 Silver
	3. Contoso 4GB Portable MP3 Player M450 White	3. MGS Age of Empires III: The Asian Dynasties M180	3. MGS Age of Empires III: The Asian Dynasties M180	3. MGS Age of Empires III: The Asian Dynasties M180
	4. Contoso 4GB Portable MP3 Player M450 White	4. MGS Age of Empires III: The Asian Dynasties M180	4. MGS Age of Empires III: The Asian Dynasties M180	4. MGS Age of Empires III: The Asian Dynasties M180
NCFO	1. Contoso Washer & Dryer 25.5in M255 Green	1. NT Wireless Bluetooth Stereo Headphones M402 Green	1. MGS Dungeon Siege: Legends of Aranna M330	1. Contoso Washer & Dryer 25.5in M255 Green
	2. Contoso 4GB Portable MP3 Player M450 Black	2. NT Wireless Bluetooth Stereo Headphones M402 Green	2. MGS Dal of Honor Airborne M150	2. NT Wireless Bluetooth Stereo Headphones M402 Green
	3. Litware Washer & Dryer 25.5in M350 Silver	3. Litware Washer & Dryer 25.5in M350 White	3. SV Hand Games men M30 Red	3. Contoso 4GB Portable MP3 Player M450 Black
	4. MGS Return of Arcade Anniversary Edition M390	4. Contoso Home Theater System 7.1 Channel M1700 Silver	4. NT Washer & Dryer 27in L2700 Green	4. Litware Washer & Dryer 25.5in M350 Silver

**Table 10:** Top-four recommendations (NCFO) and highly rated products for two pairs of aligned users. Evolved dataset and ontology.

$\left(u_{1},u_{2}\right)$—before user features—$\left(v_{1},v_{2}\right)$				
HRP	1. SV Hand Games men M30 Black	1. Litware Washer & Dryer 21in E214 Green	1. SV Hand Games women M40 Yellow	1. Contoso Home Theater System 2.1 Channel M1210 Brown
	2. SV Keyboard E90 White	2. MGS Gears of War 2008 M450	2. Contoso Washer & Dryer 24in M240 White	2. Contoso Home Theater System 5.1 Channel M1520 White
	3. SV Keyboard E90 White	3. MGS Gears of War 2008 M450	3. SV Hand Games women M40 Yellow	3. MGS Rise of Nations: Rise of Legends M290
	4. Contoso Washer & Dryer 25.5in M255 Silver	4. Litware Washer & Dryer 21in E214 Green	4. SV Hand Games women M40 Yellow	4. Contoso Home Theater System 5.1 Channel M1520 White
NCFO	1. Contoso Water Heater 2.6 GPM E0900 Grey	1. SV 40GB USB2.0 Portable Hard Disk E400 Silver	1. SV DVD 38 DVD Storage Binder E25 Red	1. MGS Return of Arcade Anniversary Edition M390
	2. MGS Rise of Nations: Rise of Legends M290	2. Contoso USB Cable M250 White	2. MGS Zoo Tycoon 2: Marine Mania Expansion Pack M270	2. NT Washer & Dryer 24in M2400 White
	3. Adventure Works Desktop PC1.80 ED180 Silver	3. Contoso Washer & Dryer 21in E210 White	3. MGS Zoo Tycoon2009 E170	3. Contoso Multi-line phones M30 Grey
	4. MGS Flight Simulator X Acceleration Expansion Pack M200	4. Litware Home Theater System 5.1 Channel M515 Black	4. Litware Washer & Dryer 24in M260 White	4. Contoso Home Theater System 4.1 Channel M1410 Brown
$\left(u_{1},u_{2}\right)$—**after user features**—$\left(v_{1},v_{2}\right)$				
HRP	1. SV Hand Games men M30 Black	1. Litware Washer & Dryer 21in E214 Green	1. SV Hand Games women M40 Yellow	1. Contoso Home Theater System 2.1 Channel M1210 Brown
	2. MGS Gears of War 2008 M450	2. SV Keyboard E90 White	2. Contoso Washer & Dryer 24in M240 White	2. Contoso Home Theater System 5.1 Channel M1520 White
	3. SV Keyboard E90 White	3. MGS Gears of War 2008 M450	3. SV Hand Games women M40 Yellow	3. MGS Rise of Nations: Rise of Legends M290
	4. Contoso Washer & Dryer 25.5in M255 Silver	4. Litware Washer & Dryer 21in E214 Green	4. SV Hand Games women M40 Yellow	4. Contoso Home Theater System 5.1 Channel M1520 White
NCFO	1. MGS Rise of Nations: Rise of Legends M290	1. SV 40GB USB2.0 Portable Hard Disk E400 Silver	1. Litware 14” High Velocity Floor Fan E801 Black	1. MGS Return of Arcade Anniversary Edition M390
	2. MGS Age of Empires, 2009 E182	2. Contoso USB Cable M250 White	2. Litware Washer & Dryer 24in M260 White	2. NT Washer & Dryer 24in M2400 White
	3. MGS Flight Simulator X Acceleration Expansion Pack M200	3. Contoso Washer & Dryer 21in E210 White	3. Contoso Home Theater System 4.1 Channel M1410 Silver	3. Contoso Home Theater System 4.1 Channel M1410 Brown
	4. Contoso Washer & Dryer 21in E210 Green	4. Litware Home Theater System 5.1 Channel M515 Black	4. SV DVD 9-Inch Player Portable M300 Silver	4. NT Washer & Dryer 21in E2100 Green

## Data Availability

The data used for this research is publicly available at https://www.microsoft.com/en-us/download/details.aspx?id=18279.
